# Modular engineering to increase intracellular NAD(H/^+^) promotes rate of extracellular electron transfer of *Shewanella oneidensis*

**DOI:** 10.1038/s41467-018-05995-8

**Published:** 2018-09-07

**Authors:** Feng Li, Yuan-Xiu Li, Ying-Xiu Cao, Lei Wang, Chen-Guang Liu, Liang Shi, Hao Song

**Affiliations:** 1 Key Laboratory of Systems Bioengineering (Ministry of Education), SynBio Research Platform, Collaborative Innovation Centre of Chemical Science and Engineering, School of Chemical Engineering and Technology, Tianjin University, Tianjin, 300072 PR China; 2 State Key Laboratory of Marine Resource Utilization in South China Sea, College of Information Science & Technology, Hainan University, Haikou, 570228 PR China; 3 State Key Laboratory of Microbial Metabolism, School of Life Sciences and Biotechnology, Shanghai Jiao Tong University, Shanghai, 200240 PR China; 4 Department of Biological Sciences and Technology, School of Environmental Studies, China University of Geoscience in Wuhan, Wuhan, 430074 Hubei PR China

## Abstract

The slow rate of extracellular electron transfer (EET) of electroactive microorganisms remains a primary bottleneck that restricts the practical applications of bioelectrochemical systems. Intracellular NAD(H/^+^) (i.e., the total level of NADH and NAD^+^) is a crucial source of the intracellular electron pool from which intracellular electrons are transferred to extracellular electron acceptors via EET pathways. However, how the total level of intracellular NAD(H/^+^) impacts the EET rate in *Shewanella oneidensis* has not been established. Here, we use a modular synthetic biology strategy to redirect metabolic flux towards NAD^+^ biosynthesis via three modules: de novo, salvage, and universal biosynthesis modules in *S. oneidensis* MR-1. The results demonstrate that an increase in intracellular NAD(H/^+^) results in the transfer of more electrons from the increased oxidation of the electron donor to the EET pathways of *S. oneidensis*, thereby enhancing intracellular electron flux and the EET rate.

## Abstract (web summary)

A bottleneck for the application of bioelectrochemical systems is the slow rate of extracellular electron transfer. Here the authors use a synthetic biology approach to redirect metabolic flux to NAD^+^ biosynthesis, which enhances the intracellular electron flux and the extracellular electron transfer rate.

## Introduction

Extracellular electron transfer (EET) of electroactive microorganisms involves bi-directional electron flow and exchange between intracellular and extracellular redox-active electron donors and acceptors^1–3^. EET underlies a number of bio-electrochemical systems (BES) for many environments and energy applications^4,5^, including microbial fuel cells (MFCs) for simultaneous biodegradation of organic wastes and bioelectricity harvest^6,7^, microbial electrolysis cells (MEC) for hydrogen production^8^, microbial desalination cells (MDC) for seawater desalination^9^, unbalanced electro-fermentation for production of biofuels^10–12^, and microbial electrosynthesis (MES) for production of valuable chemicals and biofuels from electro-reduction of CO_2_^13–17^. However, the low rate of EET remains a crucial bottleneck preventing the use of BES in industrial applications. Many efforts have been made to elucidate the fundamental molecular mechanisms of EET^18,19^ and to optimize the rate of EET from the perspectives of microbial synthetic biology^20–22^, electrode materials^23,24^, and bio-electrochemical reactor design^25^.

*Shewanella oneidensis* MR-1, one of the most well-studied metal-reducing exoelectrogens^26,27^, is capable of conducting EET to enable metal reduction or power generation in MFCs. The fundamental molecular mechanisms of EET of *S. oneidensis* have been extensively studied and elucidated in the recent decade^28–32^. Electrons derived from the oxidation of carbon sources and electron donor (i.e., lactate) by *S. oneidensis* MR-1 flow through an intracellular electron transfer pathway, namely, from NADH (a typical carrier of intracellular electrons) through intracellular menaquinol pools and a metal-reducing (Mtr) pathway, including a cytoplasmic membrane *c*-type cytochrome (*c*-Cyt) CymA, the periplasmic *c*-Cyts STC and FccA, and the outer membrane “porin-cytochrome” (OmcA-MtrCAB) to the outer membrane^33,34^, which subsequently transfer to the extracellular electron acceptors (e.g., metal oxides or electrodes) via the contacted-based EET pathway based on *c*-Cyts^35^ and soluble electron shuttle-mediated EET pathways based on flavins as electron shuttles^31,36^ or bound cofactors for MtrC and OmcA^37,38^. Based on these two underlying EET mechanisms, a number of synthetic biology strategies have been developed to enhance the rate of EET in *S. oneidensis*. For example, a synthetic riboflavin biosynthesis pathway from *Bacillus subtilis* was incorporated into *S. oneidensis*, resulting in a significant increase in secreted riboflavin and a subsequently improved EET rate^39,40^. Cyclic-di-GMP (a second messenger) was overexpressed in *S. oneidensis* to promote electroactive biofilm formation and the EET rate^41^. These approaches to promote EET rates essentially focus on the EET pathways between bacteria and electrodes; however, it remains unclear whether manipulation of the intracellular electron pool has any effect on the EET rate.

Nicotinamide adenine dinucleotide (NAD^+^) and its reduced form NADH are essential cofactors for the metabolism of microorganisms^42–46^, which are also hypothesized to be the intracellular electron pool for EET, enabling bioelectricity production of exoelectrogens. Previous efforts focused on increasing the intracellular [NADH]/[NAD^+^] ratio by means of metabolic engineering methods, thus enhancing the EET rate. For example, heterogeneous expression of formate dehydrogenase enabled enhanced NADH regeneration in *Clostridium ljungdahlii* to manipulate the [NADH]/[NAD^+^] ratio, thereby increasing the release of intracellular electrons and power generation^47^. We recently adopted a metabolic engineering strategy to engineer and drive the metabolic flux towards the enhancement of intracellular NADH regeneration and the [NADH]/[NAD^+^] ratio in *S. oneidensis*, which subsequently increased the EET rate^48^. In addition to the regulation of the intracellular [NADH]/[NAD^+^] ratio, increasing the total intracellular level of NAD(H/^+^) (i.e., total NADH and NAD^+^) via enhancing NAD^+^ de novo biosynthesis is another method to increase the EET rate that has been largely neglected in the past.

We herein develop a modular synthetic biology approach to increase de novo biosynthesis of the total endogenous NAD(H/^+^) pool in *S. oneidensis* MR-1 to explore its effect on bioelectricity production and the EET rate. According to genomic and bioinformatic studies^49–52^, the network architecture of NAD^+^ biosynthesis in *S. oneidensis* MR-1 can be categorized into three modules: the de novo pathway (Module 1), the salvage pathway (Module 2), and the universal biosynthesis pathway (Module 3). We systematically investigate twelve selected genes in the three modules and subsequently identify the five most crucial genes (i.e., *ycel*, *pncB*, *nadM*, nadD*, and nadE*) responsible for NAD^+^ biosynthesis. Upon assemblage of these five genes, metabolic flux is redirected to NAD^+^ biosynthesis, leading to a 2.1-fold increase in the total intracellular NAD(H/^+^) level in *S. oneidensis*. The EET rate of the engineered strain is characterized by the generation of electricity in MFCs, the maximum power density of which is increased by 4.4-fold from 30.2 ± 3.4 mW m^−2^ (WT) to 162.8 ± 5.6 mW m^−2^ (the recombinant *S. oneidensis*) compared with that of the wild-type (WT) *S. oneidensis* MR-1. In addition, the Coulomb efficiency is increased by 1.5-fold from 8.6% (WT) to 21.7% (the recombinant SN5 strain). Our results suggest that the total NAD(H/^+^) is a crucial intracellular electron pool, and an increase in this pool could result in the transfer of more electrons from increased oxidation of the electron donor (i.e., lactate) to the EET pathways of *S. oneidensis*, thereby enhancing intracellular electron flux and the EET rate. Our study also shows that synthetic biology strategies to increase the intracellular NAD(H/^+^) level are of great value in increasing the EET rate of exoelectrogens.

## Results

### Modular design strategy to enhance NAD^+^ biosynthesis

**Fig. 1:**
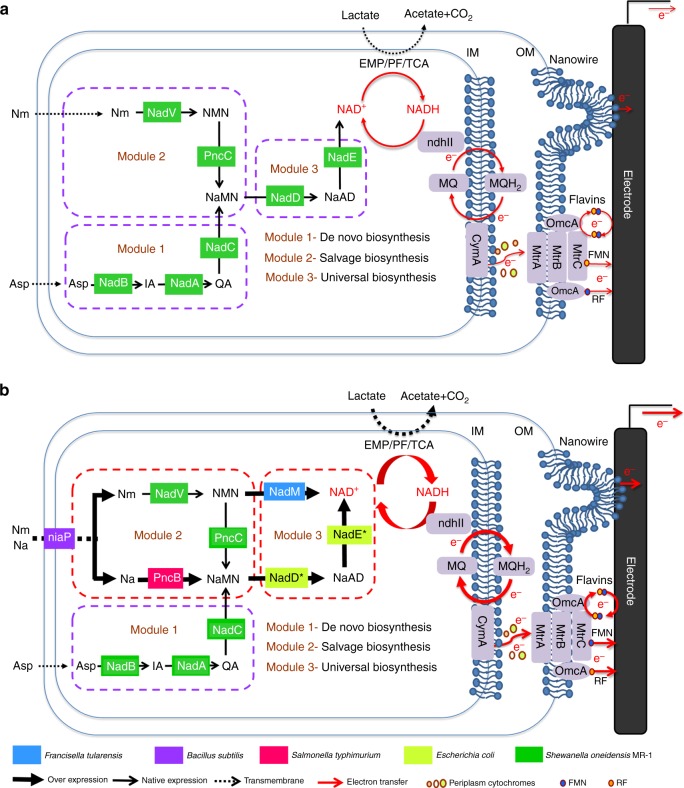
Schematic of modular design to enhance NAD^+^ biosynthesis and EET rate in *S. oneidensis* MR-1. **a** The NAD^+^ biosynthesis pathway of the wild-type *S. oneidensis* MR-1 (as revealed by genomic studies) is categorized into three modules. Module 1 involves de novo biosynthesis by assimilating L-aspartate (L-Asp). Module 2 involves salvage biosynthesis from the precursors Na (nicotinic acid) and Nm (nicotinamide). Module 3 involves universal biosynthesis from a common portion of the de novo and salvage biosynthesis pathways. **b** Recombinant *S. oneidensis* SN5 harboring five homogeneously and heterogeneously introduced genes (*ycel*, *pncB*, *nadM*, nadD*, and nadE*) to enhance NAD^+^ biosynthesis. Specifically, in Module 2, to enable simultaneously assimilation and metabolism of Na and Nm, the gene *ycel* encoding a bifunctional niaP transporter for Na and Nm from *Bacillus subtilis* and the gene *pncB* encoding nicotinate phosphoribosyltransferase from *Salmonella typhimurium* were heterogeneously expressed. In Module 3, the gene *nadM* encoding an NMN adenylyltransferase from *Francisella tularensis* was efficient in EET enhancement. Additionally, the gene nadE* (encoding NAD^+^ synthetase) and nadD* (encoding nicotinate mononucleotide adenylyltransferase) from *Escherichia coli* were confirmed to be involved in promoting the EET rate. The enzymes are represented by the corresponding protein (in rectangles on the arrows)—NadA: quinolinate synthetase, NadB: L-aspartate oxidase, NadC: quinolinate phosphoribosyltransferase, NadD*: NaMN adenylyltransferase, NadE*: NAD synthetase, NadM: NMN adenyltransferase, NadV: Nm phosphoribosyltransferase, PncB: Na phosphoribosyltransferase, PncC: Nm mononucleotide deamidase, NadM: NMN adenylyltransferase, niaP: Na and Nm transporter. Abbreviations: Asp aspartate, IA α-iminosuccinate, Qa quinolinic acid, Na nicotinic acid, Nm nicotinamide, NMN nicotinamide mononucleotide, NaMN nicotinic acid mononucleotide, NaAD nicotinic acid adenine dinucleotide, MQ methyl naphthoquinone, IM inner membrane, OM outer membrane, CymA inner membrane tetraheme *c*-Cyts, MtrA periplasmic decaheme *c*-Cyts, MtrB β-barrel trans-OM protein, MtrC and OmcA two OM decaheme *c*-Cyts, EMP Embden–Meyerhof–Parnas pathway, TCA tricarboxylic acid cycle, PF pyruvate fermentation, RF riboflavin, FMN flavin mononucleotide. The thick and thin arrows represent overexpression and native expression of genes, respectively. The dotted arrows represent the transmembrane transmission of metabolites

The microbial NAD^+^ biosynthesis pathways in microorganisms can be modularized into three categories, i.e., de novo pathways with L-aspartate (L-Asp) or tryptophan as the precursor (Module 1); salvage pathways with nicotinamide (Nm) or nicotinic acid (Na) as the precursor, respectively (Module 2)^51,52^; and a common portion of these two biosynthesis pathways are generally referred to as the universal biosynthesis pathway (Module 3). According to previous studies on the comparative genomics of the WT *S. oneidensis* MR-1^49,51^, its NAD^+^ biosynthesis mainly utilizes two pathways for the assimilation of precursors, i.e., the de novo synthesis pathway for assimilating L-Asp (Module 1) and the salvage synthesis pathway for utilizing Nm (Module 2), and one pathway for directly synthesizing NAD^+^, i.e., the universal biosynthesis pathway for synthesizing NAD^+^ (Module 3)^49^ (Fig. 1a).

To establish the relationship between total NAD(H/^+^) and the EET rate, a modular synthetic biology strategy was developed to increase intracellular NAD(H/^+^) production in the engineered *S. oneidensis* MR-1 (Fig. 1b). We systematically investigated potential dominating genes in the metabolic pathways to determine the genetic programming targets that were mostly responsible for NAD^+^ biosynthesis. First, the de novo biosynthesis pathway (Module 1, Fig. 1a) represents the fundamental pathway to assimilate the precursor (L-Asp) for NAD^+^ synthesis in *S. oneidensis* MR-1, which was engineered for enhanced L-Asp assimilation (Fig. 1b). Second, *S. oneidensis* MR-1 can assimilate Nm as a precursor. However, *S. oneidensis* MR-1 is incapable of utilizing Na as a precursor given its lack of the Na transporter and the nicotinate phosphoribosyltransferase that convert Na to nicotinic acid mononucleotide (NaMN)^49^ (Fig. 1a). It is thus hypothesized that programming the Na assimilation and Nm metabolic pathways could synergistically drive the metabolic flux from Nm and Na towards NAD^+^ (Fig. 1b). Furthermore, instead of choosing a shortcut and direct transformation pathway from nicotinamide mononucleotide (NMN) to NAD^+^ (such as that in *Francisella tularensis*) in the salvage biosynthesis pathway (Module 2)^53,54^, *S. oneidensis* MR-1 utilizes a detoured pathway from NMN to NaMN and nicotinic acid adenine dinucleotide (NaAD) and subsequently to NAD^+^ (Fig. 1a) through the salvage and universal biosynthesis pathways to synthesize NAD^+^, which may cause inefficiency in NAD^+^ biosynthesis. We thus hypothesized the exogenous introduction of a direct conversion pathway by incorporating the *nadM* gene, which would establish a bridge to directly convert NMN to NAD^+^ in the newly programmed universal pathway (Module 3, Fig. 1b). Finally, the universal biosynthesis pathway (Module 3, Fig. 1a), as the only “single-log bridge” for the entire NAD^+^ biosynthesis pathway, also represents a common portion of the de novo and the salvage biosynthesis pathways. Thus, this pathway would be further engineered to increase the metabolic flux towards NAD^+^ biosynthesis (Fig. 1b).

### Optimization of the de novo biosynthesis pathway

**Fig. 2:**
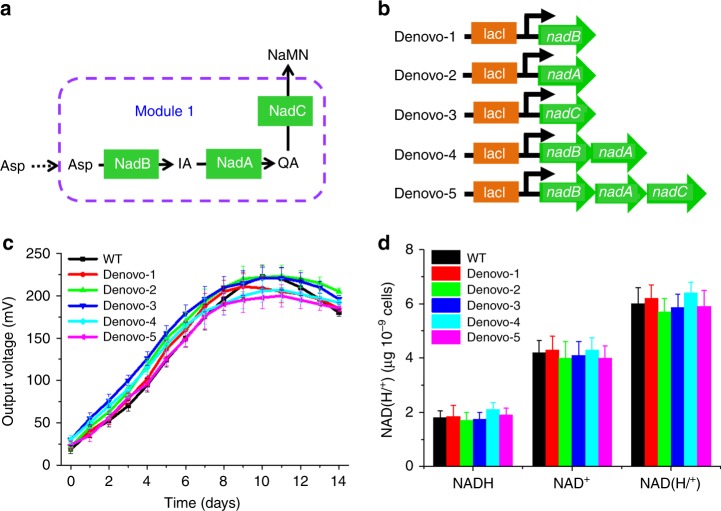
Optimization of the de novo biosynthesis pathway (Module 1) to enhance the extracellular electron transfer (EET) rate. **a** A depiction of the de novo biosynthesis pathway of NAD^+^ (Module 1). **b** Plasmids that included the assemblage of genes (*nadA*, *nadC*, and *nadB*) in Module 1. **c** Voltage output in microbial fuel cells (MFCs) of the above *S. oneidensis* recombinant strains. **d** Quantitative measurements of intracellular NAD^+^, NADH, and total NAD(H/^+^) levels of these strains under anaerobic cultures. The error bars represent the standard deviation from three independent experiments

Enhancement of the precursor supply is a common strategy to increase the flux towards the desired products in metabolic engineering^55^. Thus, to enhance de novo biosynthesis (Module 1), we overexpressed the three endogenous genes (*nadA*, *nadB*, and *nadC*)^49,51^ individually and in combination in *S. oneidensis* MR-1 (Fig. 2a) via the construction of five recombinant *S. oneidensis* strains (Fig. 2b). Although RT-qPCR and SDS-PAGE results demonstrated that the expression levels of the genes (*nadA*, *nadB*, and *nadC*) introduced in the de novo biosynthesis pathway were increased at both transcriptional and translational levels (Supplementary Fig. 1a and 1b), we found that the overexpression of these genes in Module 1 had a negligible impact on electricity production in MFCs (Fig. 2c). This result suggested that metabolic flux from Asp to NaMN was not limiting in the WT *S. oneidensis*.

Furthermore, we measured the intracellular levels of NADH and NAD^+^ (Fig. 2d) and found that the overexpression of the three genes (*nadA*, *nadB*, and *nadC*) in Module 1 had a negligible impact on the intracellular levels of both NADH and NAD^+^. Thus, the intracellular electron pool in *S. oneidensis* MR-1 was independent upon the genes’ expression levels in the de novo NAD^+^ biosynthesis pathway under the conditions tested.

### Optimization of the salvage biosynthesis pathway

Most literatures on the NAD^+^ salvage pathways had focused on the utilization of two salvageable pyridine bases, namely, Na and Nm^49–51^. In some bacteria, exogenous Na or Nm is recognized as the precursor for NAD^+^ synthesis in the salvage biosynthesis pathway^52^. Previous studies found that *S. oneidensis* MR-1 could only assimilate Nm^49^. However, the lack of the Na transporter and the nicotinate phosphoribosyltransferase converting Na to NaMN makes *S. oneidensis* MR-1 unable to utilize exogenous Na.

**Fig. 3:**
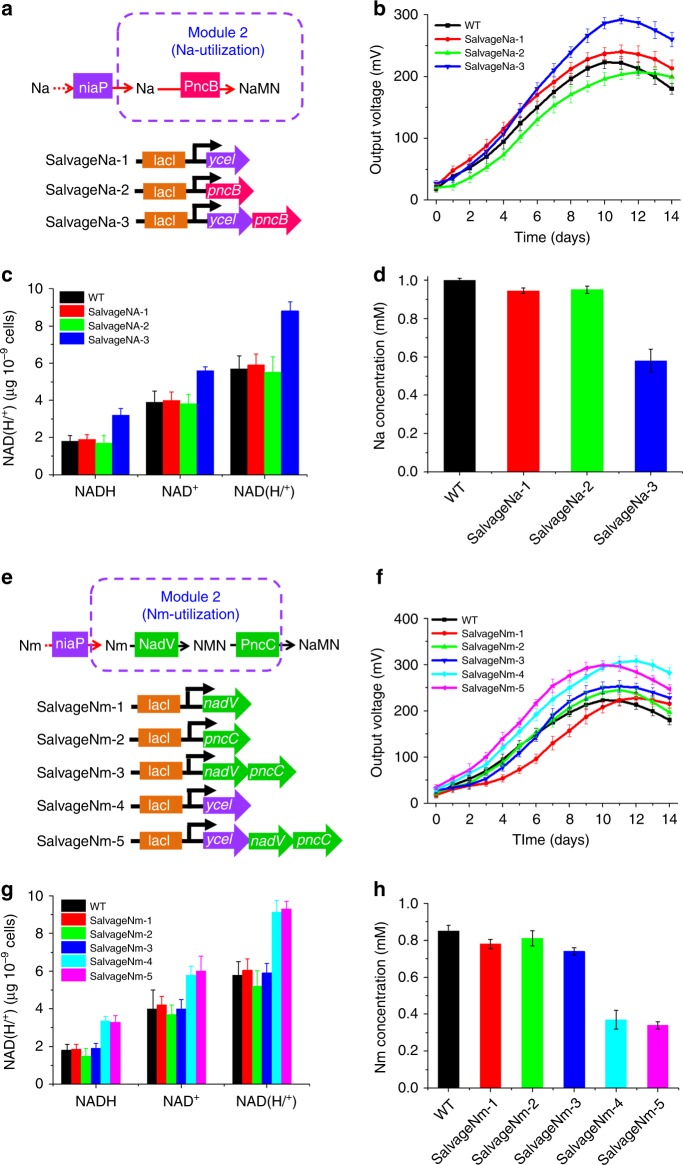
Optimization of the salvage biosynthesis pathway (Module 2) to facilitate nicotinic acid (Na) and nicotinamide (Nm) utilization. **a** A depiction of the engineered Na salvage biosynthesis pathway for NAD^+^ biosynthesis (Module 2). Plasmids that included the assemblage of genes (*pncB* and *ycel*) in the Na-utilization pathway of Module 2. **b** Voltage output in MFCs of the above *S. oneidensis* recombinant strains supplemented with 1 mM Na. **c** Quantitative measurements of the intracellular NAD^+^, NADH, and total NAD(H/^+^) levels of these strains under anaerobic cultures. **d** Na concentrations of the recombinant strains in the MFC anolytes. **e** Optimization of the salvage biosynthesis pathway (Module 2) of the Nm-utilization pathway. A depiction of the engineered Nm salvage biosynthesis pathway (Module 2). Plasmids that included the assemblage of genes (*nadV*, *pncC*, and *ycel*) in the Nm-utilization pathway of Module 2. **f** Voltage output in MFCs of the above *S. oneidensis* recombinant strains supplemented with 1 mM Nm. **g** Quantitative measurements of the intracellular NAD^+^, NADH, and total NAD(H/^+^) levels of these strains under anaerobic cultures. **h** Nm concentrations of the recombinant strains in the MFCs anolytes

A previous study demonstrated that the gene *ycel* from the Gram-positive *B. subtilis* could be successfully expressed in Gram-negative *Escherichia coli*^56^, which inspired us to express *ycel* in Gram-negative *S. oneidensis*. In addition, the overexpression of the gene *pncB* encoding nicotinate phosphoribosyltransferase from *Salmonella typhimurium* increased the total level of NAD^+^ in *E. coli*^43^. Therefore, to improve the salvage biosynthesis pathway in *S. oneidensis*, we reconstructed the Na-utilization pathway and strengthened the endogenous Nm-utilization pathway in *S. oneidensis*. To enable *S. oneidensis* MR-1 to assimilate Na, the Na-utilization pathway including the gene *ycel* from *B. subtilis* encoding a bifunctional niaP transporter for both Na and Nm^50,51,56^ and the gene *pncB* encoding the nicotinate phosphoribosyltransferase of *S. typhimurium* to convert Na to NaMN were constructed in the recombinant *S. oneidensis*. The resulting plasmids and strains are listed in Fig. 3a.

Among these strains, SalvageNa-3 possessed the synthetic Na-utilization pathway and produced a maximum voltage of 295.0 ± 9.4 mV (*n* = 3). This value was higher than that of the WT strain (223.7 ± 10.2 mV) (Fig. 3b), demonstrating that the introduction of the synthetic Na metabolic pathway could significantly enhance the EET rate. We further measured intracellular NAD(H/^+^) levels under anaerobic culturing conditions. The results showed that the intracellular NADH, NAD^+^, and NAD(H/^+^) levels in SalvageNa-3 were increased by 0.78-, 0.44-, and 0.54-fold higher than those of the WT, respectively (Fig. 3c), suggesting that the observed EET improvements were most likely attributed to the increased intracellular levels of NAD(H/^+^). In addition, the Na consumption of SalvageNa-3 exhibited greater superiority than did that of other strains (Fig. 3d). The increases in the transcriptional and translational levels of the genes (*ycel* and *pncB*) were also consistent with the increase in the level of total intracellular NAD(H/^+^), the consumption of Na, and the output voltage (Supplementary Fig. 1c and 1d). Therefore, these results demonstrated that reconstructing the Na-utilizing pathway in salvage biosynthesis enabled the utilization of exogenous Na as the precursor, thus enhancing the intracellular electron pool, i.e., the level of NAD(H/^+^) in *S. oneidensis*.

To further explore the feasibility of reinforcing Nm uptake to enhance NAD^+^ synthesis as another branch of the salvage biosynthesis (i.e., the Nm-utilization pathway), we further overexpressed the endogenous gene *nadV* encoding a Nm phosphoribosyltransferase^49,51^ and *pncC* encoding the Nm mononucleotide deamidase^49^ individually or in combination in *S. oneidensis* MR-1 (Fig. 3e), yielding five strains (Fig. 3e). MFC measurements of these strains revealed a negligible difference between the WT and SalvageNm-1, -2, and -3 strains (Fig. 3f). In contrast, SalvageNm-4 overexpressing *ycel* and SalvageNm-5 overexpressing the entire Nm-utilization pathway (i.e., simultaneously incorporating the genes *ycel*, *nadV*, and *pncC*) exhibited superior output voltages of 308.2 ± 11.2 and 301 ± 9.4 mV (*n* = 3), respectively, compared with those of the WT (Fig. 3f).

The NAD(H/^+^) measurements revealed that the intracellular NAD(H/^+^) level was significantly increased in SalvageNm-4 and -5 (Fig. 3g), which also exhibited a greater superiority in Nm compared with that of the WT (as shown in Fig. 3h). Although the RT-qPCR and SDS-PAGE results demonstrated the genes (*nadV* and *pncC*) overexpressed in the Nm-utilization pathway were increased at both the transcriptional and the translational levels in the recombinant strains SalvageNm-1, -2, and -3 (Supplementary Fig. 1c and 1d), overexpressing the genes *nadV* and *pncC* individually or in combination had minimal impact on electricity production, NAD(H/^+^) synthesis, and Nm consumption. These results suggested that overexpression of the bifunctional niaP transporter for Na and Nm could accelerate uptake of Na or Nm, improving NAD^+^ biosynthesis and consequently the EET rate. In summary, the genes *ycel* and *pncB* in the salvage pathway (Module 2) were selected for the subsequent modular assembly to promote the EET rate.

### Optimization of the universal biosynthesis pathway

The universal biosynthesis pathway (Module 3) is a common part of the de novo and the salvage biosynthesis pathways and the only “single-log bridge” for transforming the intermediates NaMN to NAD^+^ in most microorganisms. Thus, to efficiently drive the precursors (L-Asp, Na, and Nm) towards NAD^+^ biosynthesis, we not only reinforced the native metabolic flux from NaMN to NAD^+^ but also reconstructed the new metabolic pathway directly from NMN to NAD^+^ (Fig. 1b).

**Fig. 4:**
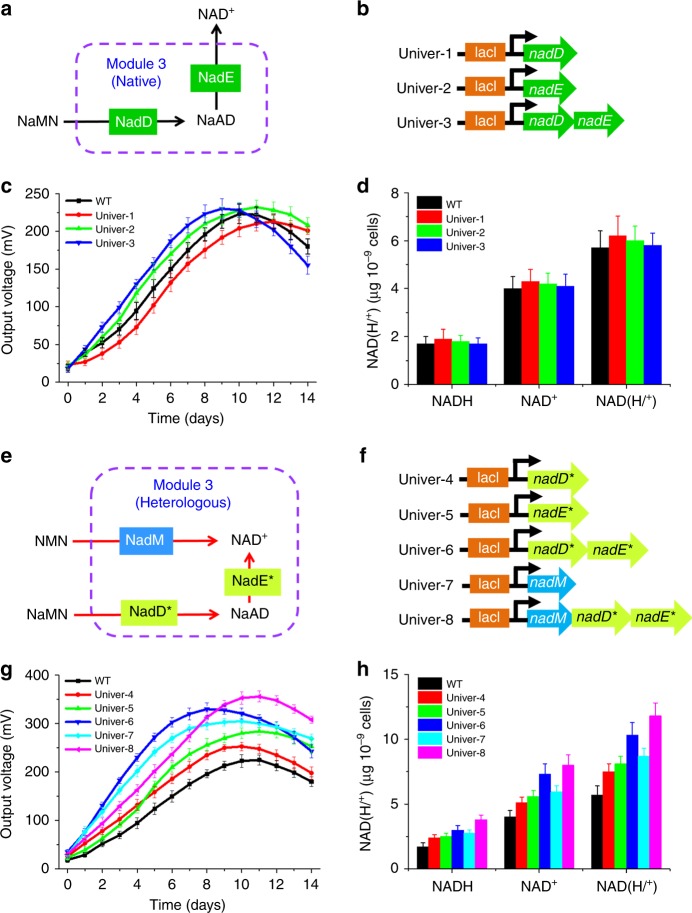
Optimization of the universal biosynthesis pathway of *S. oneidensis* (Module 3). **a** A depiction of the overexpressed genes in the universal biosynthesis pathway (Module 3). **b** Plasmids that included the assemblage of genes (*nadD* and *nadE*) in the universal biosynthesis pathway of Module 3. **c** Voltage output in MFCs of the above *S. oneidensis* recombinant strains. **d** Quantitative measurements of the intracellular NAD^+^, NADH, and total NAD(H/^+^) levels of these strains under anaerobic cultures. **e** Rewiring the universal biosynthesis pathway by introducing a heterogeneous *nadM* pathway in addition to the native universal *nadD*–*nadE* pathway (Module 3). **f** A depict of the overexpressed heterogeneous *nadM* gene in the native universal biosynthesis pathway (Module 3). Plasmids that included the assemblage of genes (*nadM*, *nadD*, and *nadE*) in the universal biosynthesis pathway of Module 3. **g** Voltage output in MFCs of the above *S. oneidensis* recombinant strains, respectively. **h** Quantitative measurements of the intracellular NAD^+^, NADH, and total NAD(H/^+^) levels of these strains under anaerobic cultures

To relieve the rate-limiting steps in the universal biosynthesis pathway and drive more metabolic flux from NaMN to NAD^+^, we strengthened the native universal pathway by overexpressing the native gene *nadE* encoding the NAD^+^ synthetase and the native gene *nadD* encoding the nicotinate mononucleotide adenylyltransferase^49,51^ in Module 3 (Fig. 4a), leading to three recombinant strains Univer-1, -2, and -3 (Fig. 4b). Although RT-qPCR and SDS-PAGE results demonstrated that overexpression of the genes *nadE* and *nadD* from the universal biosynthesis pathway occurred at both transcriptional and translational levels (Supplementary Fig. 1e and 1f), electricity production (Fig. 4c) and intracellular levels of NAD(H/^+^) (Fig. 4d) in the recombinant strains were essentially similar to those of the WT strains, suggesting that the overexpression of the native genes *nadE* and *nadD* in Module 3 had a negligible impact on the EET rate.

Previous experiments demonstrated that nadE^*^ and nadD^*^ overexpression improve NAD^+^ synthesis and promote the EET rate in *E. coli*^57,58^ and *Pseudomonas aeruginosa*^59^. Comparative genomic and bioinformatic analyses also demonstrated that the gene *nadM* encoding the NMN adenylyltransferase from *F. tularensis*^53^ possess dual substrate specificity towards both NaMN and NMN, which could transform NMN to NAD^+^ in *E. coli*. Thus, to further convert both of the intermediates (NaMN and NMN) to NAD^+^, we first overexpressed the heterogeneous genes nadE* and nadD* from *E. coli* to convert NaMN to NAD^+^^57,58^ in *S. oneidensis*. In addition, we constructed a shortcut pathway that directly channeled NMN from the Nm-utilizing pathway towards NAD^+^ synthesis. This shortcut pathway was constructed by heterologous expression of the NMN adenylyltransferase of *F. tularensis*, which could directly transform NMN to NAD^+^^53,54^ (Fig. 4e). Thus, the recombinant strains (Univer-4, -5, -6, -7, and -8) that overexpressed nadE*, nadD*, and *nadM* were obtained (Fig. 4f). Notably, Univer-6 with a heterogeneous universal biosynthesis pathway and Univer-7 with the *nadM* gene produced maximum voltages of 330.4 ± 8.4 and 305.2 ± 4.3 mV (*n* = 3), respectively, which were significantly increased compared with those of the WT (Fig. 4g). To further improve the output voltage, Univer-8 was constructed based on the simultaneous expression of the three genes nadE*, nadD*, and *nadM*, which produced a maximum voltage of 356.6 ± 9.4 mV (*n* = 3) (Fig. 4g).

We also examined intracellular NADH and NAD^+^ levels in these strains under anaerobic culturing conditions. NADH, NAD^+^, and NAD(H/^+^) levels in Univer-8 were increased by 0.88-, 0.90-, and 0.86-fold compared with those of the WT, respectively (Fig. 4h). The increase in the transcriptional and translational levels of the three genes nadE*, nadD*, and *nadM* enabled an increase in the level of the total NAD(H/^+^) in *S. oneidensis* and the output voltage (Supplementary Fig. 1e and 1f). Hence, the increase in the metabolic flux of the universal biosynthesis pathway towards NAD^+^ synthesis could enhance the total NAD(H/^+^) level and EET rate. Thus, we selected the genes nadE*, nadD*, and *nadM* for subsequent modular assembly.

### Modular assembly enhances intracellular NAD(H/^+^)

**Fig. 5:**
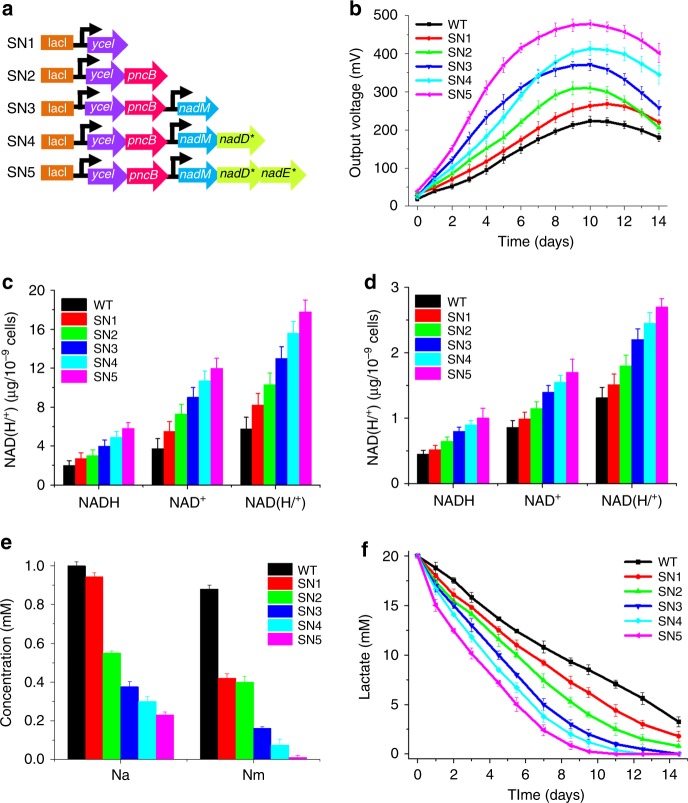
Modular assembly of the five crucial genes for enhanced NAD^+^ biosynthesis to increase the extracellular electron transfer (EET) rate. **a** A depiction of the plasmids that incorporated the modular assemblage of the five crucial genes (*ycel*, *pncB*, *nadM*, nadD*, and nadE*) identified from the three modules (Module-1, -2, and -3), which had a significant impact on NAD^+^ biosynthesis. **b** Voltage output in MFCs of the *S. oneidensis* recombinant strains programmed with the above plasmids, respectively. **c** Quantitative measurements of the intracellular NAD^+^, NADH, and total NAD(H/^+^) levels of these recombinant strains in anaerobic cultures. **d** Quantification of intracellular NAD^+^, NADH, and total NAD(H/^+^) levels of these recombinant strains in MFCs after discharge. **e** Nicotinic acid (Na) and nicotinamide (Nm) concentrations in the MFCs anolytes of the recombinant *S. oneidensis* strains. **f** Lactate consumption in the MFCs inoculated with these *S. oneidensis* recombinant strains programmed with the above plasmids SN1–SN5, respectively

The five genes (*ycel*, *pncB*, *nadM*, nadD*, and nadE*) identified from the above three modules were assembled by a number of Biobrick-ligation steps, resulting in five *S. oneidensis* recombinant strains (SN1 to SN5, Fig. 5a). All genes were induced at the full expression level by 1.0 mM isopropyl-β-d-thiogalactoside (IPTG) (Supplementary Fig. 2), and the RT-qPCR and SDS-PAGE results demonstrated that the expression of the five genes was increased at both the transcriptional and the translational levels in SN5 (Supplementary Fig. 3). As shown in Fig. 5b, the voltage output curves of MFCs with SN1 to SN5 showed that the output voltage increased with the gradual incorporation of each of the five genes. SN5 with the expression of all the five genes generated the highest output voltage, demonstrating the highest EET rate.

To determine whether the enhancement of EET originated from the increase in the intracellular electron pool, intracellular NAD(H/^+^) levels of the WT and the five recombinant strains were measured under no discharge cultures and MFC discharge conditions. The intracellular levels of NAD^+^, NADH, and NAD(H/^+^) pool increased with the introduction of each of the five genes under no discharge cultures (Fig. 5c) and MFC conditions after discharge (Fig. 5d). As shown in Fig. 5c, the intracellular NAD(H/^+^) pool size of SN5 increased by 2.1-fold compared with that of the WT under no discharge conditions. In addition, compared with that of the WT, the intracellular NAD^+^ level of SN5 increased by 2.2-fold, which consequently further promoted the level of NADH, a typical carrier of intracellular electrons, by 1.9-fold compared with that of the WT by NADH regeneration. Similarly, after discharge, NAD^+^, NADH, and NAD(H/^+^) of SN5 levels increased by 1.2-, 1.0-, and 1.1-fold, respectively, compared with those of the WT (Fig. 5d). All these results collectively demonstrated that the enhanced EET was attributed to the increase in the intracellular NAD(H/^+^) pool. In addition, we further quantified the consumption of Na and Nm under MFCs conditions and found that both the Na and Nm consumption were consistent with the increase in the total intracellular NAD(H/^+^) in MFCs (Fig. 5e). Specifically, Na was significantly consumed, and Nm was almost completely consumed in MFCs by SN5. Therefore, the results demonstrated that the increase in the metabolic flux from the precursors (Na and Nm) towards the NAD^+^ biosynthesis could substantially increase the intracellular electron pool in *S. oneidensis*.

Of note, the total NAD(H/^+^) level was considerably increased in the no discharge condition compared with that in the MFC discharge condition. Before MFC discharge, *S. oneidensis* cells were grown in a high concentration of carbon sources and electron donors (i.e., lactate), which enabled high intracellular electron pool level based on lactate digestion. Upon reaching the highest voltage in the batch mode operation of MFCs, lactate was continuously consumed until depletion (Fig. 5f), and an insufficient carbon source was not available for the regeneration of intracellular NADH. Under such conditions, excessive NAD^+^ accumulates in cells, which could disturb the intracellular homeostasis balance, including the intracellular redox state, energy metabolism, carbon flux, the cell life cycle, and microbial virulence^46^. To maintain the cellular homeostatic balance, especially redox balance, cells degrade excess NAD^+^ to ADP-ribose, ribose-5-phosphate, and phosphoribosyl pyrophosphate via NAD^+^-consuming enzymes, thus resulting in low intracellular electron pool levels^45,56^. In addition, the [NADH]/[NAD^+^] ratio was generally unaltered before and after discharge, suggesting that EET could indirectly regulate intracellular redox balance^12^. As shown in Fig. 5f, SN5 exhibited a more rapid lactate consumption rate than that of the WT. The results indicated that the increase in intracellular NAD^+^ levels enabled more rapid formation of NADH from the increased oxidation of the electron donor (lactate), thus enhancing intracellular electron flux and the EET rate.

### Electrochemical characterization

**Fig. 6:**
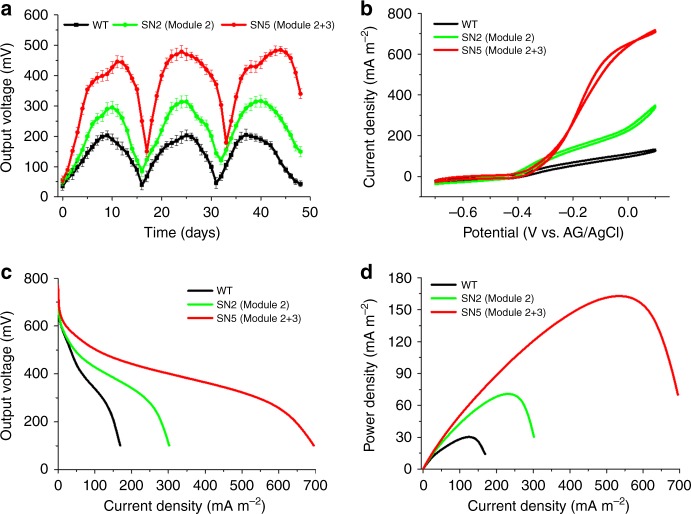
Bioelectrochemical characterization of the wild-type (WT) and two recombinant *S. oneidensis* strains (SN2 and SN5). **a** Multiple-cycle voltage output of the WT *S. oneidensis* harboring the empty vector (black line), the optimized Module 2 expressing vector (light green line), and the optimized Module 2 and Module 3 expressing vector (red line) simultaneously in MFCs. **b** Turnover cyclic voltammetry (CV) at a scan rate of 1 mV s^−1^. **c** Polarization discharge curves obtained by linear sweep voltammetry (LSV) with a scan rate of 0.1 mV s^−1^. The reduced slope of the polarization curve obtained from the recombinant *S. oneidensis* SN5 was the smallest, indicating less internal resistance. **d** Power density output curves. SN5 harboring optimized Module 2 and Module 3 obtained a maximum power density of 162.8 ± 5.6 mW m^−2^, which was greater than that of SN2 harboring Module 2 (70.8 ± 6.6 mW m^−2^) and the WT (30.2 ± 3.4 mW m^−2^)

The output voltage of MFCs with a multi-cycle operation revealed that SN5 exhibited stable power generation with a maximum voltage of 488.4 ± 12.4 mV (*n* = 3), which was much higher than that of the control strain (223.7 ± 10.2 mV (*n* = 3)) (Fig. 6a). The Coulomb efficiency was increased by 1.5-fold from 8.6% (WT) to 21.7% (SN5), suggesting that SN5 could release more electrons to the electrode than that of the WT.

Cyclic voltammetry (CV) at a low scan rate (1 mV s^−1^, allowing the establishment of pseudo-steady state redox reactions) was used to reveal redox reaction kinetics at cell–electrode interfaces. CV for the WT and SN2 with optimized Module-2 and SN5 with optimized Module-2 and -3 is presented in Fig. 6b. The result demonstrated that SN5 had the highest current density compared with that of the WT and SN2. In addition, both the WT and the recombinant *S. oneidensis* SN5 exhibited ∼52 μg cm^−2^ protein attached on the electrodes (Supplementary Fig. 4a). The average current per cell was also increased by 4.5-fold from 0.25 (WT) to 1.38 μA μg^−1^ (SN5) (Supplementary Fig. 4b). Our results showed that the main reason underlying the EET enhancement originated from the increase in the total intracellular NAD(H/^+^) level not from changes in the key components of the EET pathways, including an insignificant change in MtrCAB complex expression levels (Supplementary Fig. 5) and a 0.35-μM increase in riboflavin biosynthesis (Supplementary Fig. 6) based upon the exogenous genes programmed in SN5. In summary, the results suggested that the overall elevated EET was due to enhanced bioelectroactivity of each cell enabled by synthetic NAD^+^ pathways.

The polarization curves (output voltage vs. current density) and power output curves (power density vs. current density) were measured to further investigate the bioelectricity generation capability of MFCs inoculated with the WT and recombinant *S. oneidensis* SN2 and SN5 strains. The polarization curve reflected the potential reduction with the increase in current density when the external resistance decreased. According to the linear portion of the polarization curve (Fig. 6c), the reducing slope of polarization curves obtained from the recombinant *S. oneidensis* SN5 was smaller than that obtained from the WT and SN2, implying that the internal charge transfer resistance of the MFCs inoculated with SN5 was the smallest. The power density output curves revealed that SN5 obtained a maximum power density of 162.8 ± 5.6 mW m^−2^ (*n* = 3), which is increased by ∼4.4- and ∼1.3-fold compared with that of the WT *S. oneidensis* (30.3 ± 3.4 mW m^−2^, *n* = 3) and SN2 (70.8 ± 6.6 mW m^−2^, *n* = 3) (Fig. 6d).

## Discussion

In the last decade, extensive studies on exoelectrogens were conducted from multidisciplinary fields to elucidate fundamental mechanisms of EET. Many approaches to promote power generation primarily involved facilitating EET between bacteria and electrodes to promote the EET rate^20,21^, including broadening and strengthening substrate utilization^60^, optimizing conductive *c*-type cytochromes systems^61^, promoting electron shuttle biosynthesis and transportation^39,40,62^, and constructing electroactive biofilms^41^. However, whether the intracellular electrons pool had any effect on the EET rate remained uncharacterized. For exoelectrogens, the total NAD(H/^+^) level was the major intracellular electron pool for the EET and bioelectricity production in addition to serving as an essential cofactor for cellular metabolism.

Recent studies focused on using metabolic engineering approaches to increase the intracellular ratio of [NADH]/[NAD^+^] to enhance the EET rate. For example, heterogeneous expression of the *fdh* gene encoding formate dehydrogenase to enhance NADH regeneration and to increase the [NADH]/[NAD^+^] ratio was achieved in *C. ljungdahlii*^47^. A modular metabolic engineering strategy was used to engineer *S. oneidensis* to drive metabolic flux towards the enhancement of intracellular NADH regeneration, in which three endogenous genes (*gapA2*, *mdh*, and *pflB*) and an exogenous gene fdh* from *Candida boidinii* were identified and assembled to increase the NADH regeneration, leading to a ∼3.0-fold increase in the maximum power output^48^. In addition, the NADH dehydrogenases show a substantially preferable affinity for NADH compared with that for NAD^+^^63^, which may alter the ratio of quinol/quinones in the upstream EET pathway and thus significantly promote the EET rate. These two studies focused on regulating the [NADH]/[NAD^+^] ratio to enhance NADH regeneration and subsequently increase the EET rate (represented by the maximum power output). In addition to the methods of regulating the intracellular [NADH]/[NAD^+^] ratio to increase the intracellular NADH level, another method involves programming NAD^+^ de novo biosynthesis, in which the synthetic NAD^+^ can be further reduced to NADH by the NAD^+^-dependent dehydrogenases in the central carbon metabolism of *S. oneidensis* MR-1^48^, thus increasing the intracellular NADH level and the EET rate.

Although the idea that NADH is important for EET was thoughtfully reviewed^20,21^, the extent to which the intracellular NAD^+^ level could be promoted in *S. oneidensis* and how such increases in the total NAD(H/^+^) levels could influence the EET rate remain unclear. To address these questions, we are the first to develop a synthetic biological design approach to systematically reconstruct genetic pathways, which enabled the redirection of the metabolic flux towards NAD^+^ de novo biosynthesis for enhanced NAD^+^ biosynthesis in *S. oneidensis*.

First, to enable the simultaneous assimilation and metabolism of Na and Nm (the precursors for the NAD^+^ biosynthesis) in the salvage biosynthesis module, the gene *ycel* encoding a bifunctional niaP transporter for Na and Nm and the gene *pncB* encoding a nicotinate phosphoribosyltransferase were selected from *B. subtilis* and *S. typhimurium*, respectively. A comparative genomic analysis showed that the transcription regulator NiaR could regulate many genes encoding NAD^+^ synthesizing enzymes in *B. subtilis*^56^. Among these genes, the gene *ycel* encoding a bifunctional niaP transporter is responsible for the intake of Na and Nm based on its knockout in the *B. subtilis* genome or heterologous expression in *E. coli*^56^. This finding demonstrated that the gene *ycel* from Gram-positive *B. subtilis* could be successfully expressed in Gram-negative *E. coil*, which also inspired us to choose the gene *ycel* in Gram-positive *Bacillus* to encode the Na and Nm transporter in Gram-negative *S. oneidensis*. In addition, previous research showed that the overexpression of the *pncB* gene encoding a nicotinate phosphoribosyltransferase from *S. typhimurium* could increase total NAD^+^ levels from Na in *E. coli*^43^. Thus, to construct a complete Na-utilization pathway in *S. oneidensis*, we selected the genes *ycel* (originated from *B. subtilis*) and *pncB* (originated from *S. typhimurium*) to engineer the salvage pathway (Module 2) to promote NAD^+^ biosynthesis in *S. oneidensis*.

Second, to enable a shortcut to directly channel NMN from the Nm-utilizing pathway to NAD^+^ biosynthesis in the universal module (Module 3, heterologous), the gene *nadM* encoding an NMN adenylyltransferase was selected from *F. tularensis*. Recently, comparative genome analysis and bioinformatics prediction showed that the gene *nadM* encoding NMN adenylyltransferase from *F. tularensis*^53^ and *Acinetobacter baylyi*^50^ possess dual substrate specificity towards both Na and Nm mononucleotide. We utilized the *nadM* gene from *F. tularensis* for subsequent modular engineering, improving the biosynthesis of total NAD(H^/+^) and promoting EET efficiency in *S. oneidensis*.

Third, to relieve the rate-limiting steps in the universal biosynthesis pathway and drive more metabolic flux from NaMN to NAD^+^, the genes nadE* and nadD* were selected from *E. coli* to strengthen the conversion of NaMN to NAD^+^. Previous experiments revealed that nadE* and nadD* overexpression improves the synthesis of NAD^+^ and promotes EET efficiency in *E. coli*^57,58^ and *P. aeruginosa*^59^. We further verified here that the heterologous expression of nadE* and nadD* from *E. coli* enabled a higher voltage output in *S. oneidensis* than that of its native genes *nadE* and *nadD*. Simultaneous expression of the two genes nadE* and nadD* exhibited a superior performance in power generation compared with that of individual expression. Thus, we selected the genes nadE* and nadD* from *E. coli* for the subsequent modular assembly to programme *S. oneidensis*.

Finally, using a systematically study on the genes that were mostly responsible for NAD^+^ biosynthesis in each of the three modules (Figs. 2–6), five critical genes (*ycel*, *pncB*, *nadM*, nadD*, and nadE*) from the three modules were identified and sequentially assembled for overexpression in *S. oneidensis* (Fig. 5a). The resulting recombinant strain SN5 with the complete incorporation of all five genes generated the highest power density output in MFCs (162.8 ± 5.6 mW m^−2^), which was ∼4.4-fold increase compared with that of the WT *S. oneidensis* MR-1 (30.2 ± 3.4 mW m^−2^) (Fig. 6d). In addition, the Coulomb efficiency was increased by 1.5-fold from 8.6% (the WT *S. oneidensis*) to 21.7% (recombinant *S. oneidensis* SN5). The average current per cell was accordingly increased by 4.5-fold from 0.25 (WT) to 1.38 μA μg^−1^ (SN5). The underlying mechanism of the promotion of EET rate could be attributed to the increase in the intracellular electron pool (i.e., NAD(H/^+^)), which could transfer more electrons from the increased oxidation of the electron donor (i.e., lactate) to the EET pathway of *S. oneidensis*, thereby enhancing intracellular electron flux and the EET rate.

## Methods

### In vitro gene synthesis

The information and coding sequences of nadE*and nadD* genes of *E. coli*^57,58^, *ycel* gene of *B. subtilis*^50,51^, *pncB* gene of *S. typhimurium*^43,44^, and *nadM* gene of *F. tularensis*^53,54^ were extracted from NCBI database (Supplementary Table 1) and adapted for expression in *S. oneidensis* using a java codon adaption tool (http://www.jcat.de/) to prevent blocked translation due to a shortage of tRNAs for rare codons^39^. Each gene component was synthesized as a biobrick, and restriction enzyme sites of EcoRI, XbaI, SpeI, and SbfI were avoided in the codon-optimized sequences. The optimized gene sequence was flanked by an upstream prefix (containing EcoRI and XbaI), an RBS site (BBa_B0034, iGEM) 6 bp ahead of the start codon, and a downstream suffix (containing SpeI and SbfI) (Supplementary Table 2). The designed gene sequences were synthesized in vitro and verified by Sanger sequencing (AuGCT, China).

### Plasmid construction and bacterial culture

All plasmid constructions were performed in *E. coli* Trans T1. *E. coli* strains were cultured in Luria-Bertani (LB) medium at 37 °C with 220×*g*. When needed, 50 μg ml^−1^ kanamycin was added in the culture medium for plasmid maintenance. To benefit the multigene assembly in *S. oneidensis*, a Biobrick compatible expression vector pYYDT allowing various gene expression levels upon differential IPTG induction was adopted as previously constructed in our lab^39^. Plasmids to be transformed into *S. oneidensis* MR-1 (ATCC700550) were first transformed into the plasmid donor strain *E. coli* WM3064 (auxotroph) and transferred into *S. oneidensis* by conjugation. Then, 100 μg ml^−1^ 2,6-diaminopimelic acid (DAP) was added for the growth of *E. coli* WM3064.

### BES setup

To evaluate the efficiency of EET, the overnight *S. oneidensis* culture suspension (0.5 ml) was inoculated into 50 ml fresh LB broth and incubated at 30 °C with shaking (200×*g*) until the optical density (OD_600_) of the cell culture reached ∼1.0. Then, the cells were harvested by centrifugation and washed three times with fresh M9 buffer (Supplementary Table 3). The cell pellets were subsequently re-suspended in 140 ml electrolyte (5% LB broth plus 95% M9 buffer supplemented with 20 mM lactate and 1 mM Na and Nm). Additionally, previous experiments in our lab demonstrated that IPTG had no effect on the cell physiology and EET of *S. oneidensis*. The culture medium was supplemented with 1.0 mM IPTG (optimized in Supplementary Fig. 2) and 50 μg ml^−1^ kanamycin to ensure consistent culture conditions. Dual-chamber MFCs (140 ml working volume) separated by nafion 117 membranes (DuPont Inc., USA) were used. Carbon cloth was used as the electrodes for both the anode (2.5 cm × 2.5 cm, i.e., the geometric area is 6.25 cm^2^) and cathode (2.5 cm × 3 cm). The cathodic electrolyte was composed of 50 mM K_3_[Fe(CN)_6_] in 50 mM K_2_HPO_4_ and 50 mM KH_2_PO_4_ solution. To measure the voltage generated, a 2 kΩ external resistor was connected into the external circuit of MFCs, and the MFC potential was recorded using a digital multimeter (DT9205A).

### Electrochemical analyses

CV analysis with a scan rate (1 mV s^−1^) was performed in a three-electrode configuration with an Ag/AgCl as reference electrode on a CHI 1000C multichannel potentiostat (CH Instrument, Shanghai, China). Linear sweep voltammetry (LSV) analysis with a slow scan rate (0.1 mV s^−1^) was conducted on a two-electrode mode to obtain the polarization curves to estimate the maximum power density (the potential decreased from the open circuit potential (OCP) to −0.1 V). Power density (*P*) was calculated using Eq. (1):

1 $$P{\mathrm{ = }}\frac{{VI}}{S}$$

where *V* is the output voltage; *I* is the current; and *S* is the projected area of the anode surfaces.

The Coulombic efficiency (*C*_*E*_) is defined as the ratio of the Coulombs actually recovered as current to maximum possible Coulombs if all substrate was consumed to produce current. The Coulombs actually recovered was determined by integrating the current (*I*) over a period of batch cycles (*t*)^64^. Thus, the Coulombic efficiency can be evaluated over a period of time using Eq. (2):

2 $$\begin{array}{l}C_E{\mathrm{ = }}\frac{{Coulombs\,recovered}}{{Total\,coulombs\,in\,substrate}}{\mathrm{ = }}\frac{{M_S\mathop {\int }\nolimits_0^{t_b} Idt}}{{Fb_{ES}V_{An}\Delta c}}\\ = \frac{{M_SIt_b}}{{Fb_{ES}V_{An}\Delta c}}\end{array}$$

where *M* (g mol^−1^) is the molecular weight of the substrate (here lactate), *F* is Faraday’s constant (98,485 C mol^−1^ of electrons), *I* (A) is the current, *t*_*b*_ (s) is the time period of a batch cycle, *b*_*ES*_ is the stoichiometric number of moles of electrons produced per mole of substrate (*b* = 4 when lactate was used as the substrate), *V* (l) is the volume of liquid in the anode compartment, and Δ*c* (g l^−1^) is the change of substrate concentration over a batch MFC cycle.

### Quantification of metabolites

For the quantification of Na and Nm, the samples in the anolytes were analyzed using a high-performance liquid chromatography (HPLC) system equipped with a UV detector at 263 nm. The 0.02 M KH_2_PO_4_–acetonitrile (90:10, v/v) was used as mobile phase flowing at 1.0 ml min^−1^ through the Grace Apollo C8 column (4.6 mm × 250 mm, particle size: 5 µm), which was incubated at 28 °C. Lactate in the anolytes was quantified by HPLC with an organic acid column (Aminex HPX-87H Column, 300 mm × 7.8 mm, Bio-Rad) incubated at 65 °C and a refractive index detector (Waters, Corp.). The mobile phase was 0.005 M H_2_SO_4_ at a flow rate of 0.6 ml min^−1^. Samples were centrifuged at 12,000×*g* for 1 min to remove cells and then filtered with 0.22-μm syringe filters (Nylon) before injection. For the quantification of riboflavin, the samples in the MFCs supernatant were first centrifuged (35,000×*g* for 20 min) and filtered (0.22 μm), and the eluted medium were detected by a liquid chromatography-tandem mass spectrometer (LC-MS) (Agilent LCMS-1290-6460) in positive ion mode using a Waters XBridge C8 column (2.1  mm × 100 mm; particle size: 3.5 μm).

### qRT-PCR

A bacterial total RNA extraction kit (APEXBIO, China) was used to isolate total RNA from mid-log-phase cultures according to the manufacturer’s instructions. Additionally, the GoScript reverse transcription system (Promega, USA) was used to synthesize cDNA, and Sso Advanced SYBR Green Supermix (Bio-Rad, USA) was used to perform quantitative analyses of target gene expression. The *gyrB* gene was used as the reference gene due to its relatively stable expression. The expression levels of the target genes were normalized with respect to the expression level of *gyrB*. The primers, which were used to amplify the small parts of the gene, are listed in Supplementary Table 4. The data were analyzed using the 2^−ΔΔCT^ method^65^.

### Measurement of protein levels

To evaluate protein expression levels, sodium dodecyl sulphate polyacrylamide gel electrophoresis (SDS-PAGE) was performed^66^. The cells were harvested by centrifugation at 6000×*g* for 10 min at 4 °C and washed thrice with sterile 0.01 M PBS buffer (pH 7.2–7.4). The suspended cells were disrupted by sonification on ice. The total protein or supernatant was used to perform SDS-PAGE. Slab gels of a 4% (w/v) stacking gel and a 10% (w/v) separating gel were employed for protein resolution.

### Qualification of *c*-type cytochromes

The *c*-type cytochromes were probed by using a haeme-linked peroxidase staining method^30,67^. The samples without reducing reagents or heating were the first subject to SDS-PAGE. Gels were soaked with 30% (v/v) of 3, 3′, 5, 5′-tetramethylbenzidine-H_2_O_2_ (TMBZ) methanol solution and 70% (v/v) 0.25 M sodium acetate (pH = 5.0) for 3 h in the dark. Then, H_2_O_2_ was added to a final concentration of 30 mM. After haeme-containing bands fully developed (3 min to overnight), the gel was washed with 30% (v/v) isopropanol and 70% (v/v) 0.25 M sodium acetate (pH = 5.0).

### Quantification of the intracellular NAD(H/^+^)

The cells (10 ml) were first cooled in ice bath for 20 min to retard cell metabolism, collected by centrifugation (10,000×*g* at 4 °C for 5 min) and immediately resuspended in 300 μl of 0.2 M HCl (for NAD^+^) or 0.2 M NaOH (for NADH). The suspensions were boiled for 7 min, and rapidly quenched in an ice bath. Then, 300 μl of 0.1 M HCl (for NADH) or 0.1 M NaOH (for NAD^+^) was added. Cell debris was removed by centrifugation at 10,000×*g* at 4 °C for 10 min, and the supernatant was used in a cycling assay to determine the amounts of NAD^+^ and NADH^59,68^. The cell concentration for the detection of NAD^+^ and NADH concentration was detected by plate counts on LB agar.

### Measurement of electrode-attached biomass

The electrode was placed in a 50-ml tube containing 5 ml of 0.2 M NaOH that was vortexed for 2 min and incubated in a water bath (at 96 °C for 30 min) to lyse cells. After cooling to room temperature, the extracts were tested using a bicinchoninic acid protein assay kit (Solarbio, China) according to the manufacturer’s instructions.

## Electronic supplementary material

Supplementary Information

## Data Availability

The authors declare that all data supporting the findings of this study are available within the paper and its supplementary information files. Data supporting the findings of this study are available from the corresponding author on request. Supplementary Table 1 presents the functional role and the source of the overexpressed genes in this study. Supplementary Table 2 presents the synthesized gene sequences.
