# Supplementary material for: Modular engineering to increase intracellular NAD(H/+) promotes rate of extracellular electron transfer of Shewanella oneidensis
Source: Nat Commun. 2018 Sep 7;9:3637. doi: 10.1038/s41467-018-05995-8 (PMC6128845; doi:10.1038/s41467-018-05995-8)
Supplement: Supplementary file 1 — Supplementary Information [file 41467_2018_5995_MOESM1_ESM.pdf]

Supplementary Information

Modular engineering to increase intracellular NAD(H/$^+$) promotes rate of extracellular electron transfer of *Shewanella oneidensis*

Feng Li [a ‡], Yuanxiu Li [a ‡], Yingxiu Cao [a], Lei Wang [b], Chenguang Liu [c], Liang Shi [d], Hao Song [a,*]

[a] Key Laboratory of Systems Bioengineering (Ministry of Education), SynBio Research Platform, Collaborative Innovation Centre of Chemical Science and Engineering, School of Chemical Engineering and Technology, Tianjin University, Tianjin 300072, P. R. China.

[b] State Key Laboratory of Marine Resource Utilization in South China Sea, College of Information Science & Technology, Hainan University, Haikou 570228, P. R. China.

[c] State Key Laboratory of Microbial Metabolism, School of Life Sciences and Biotechnology, Shanghai Jiao Tong University, Shanghai 200240, P. R. China.

[d] Department of Biological Sciences and Technology, School of Environmental Studies, China University of Geoscience in Wuhan, Wuhan, Hubei 430074, P. R. China.

* Corresponding author: H. Song, E-mail: hsong@tju.edu.cn; Tel: +86-18722024233

‡ Equal contribution

Module 1 - *De novo* biosynthesis

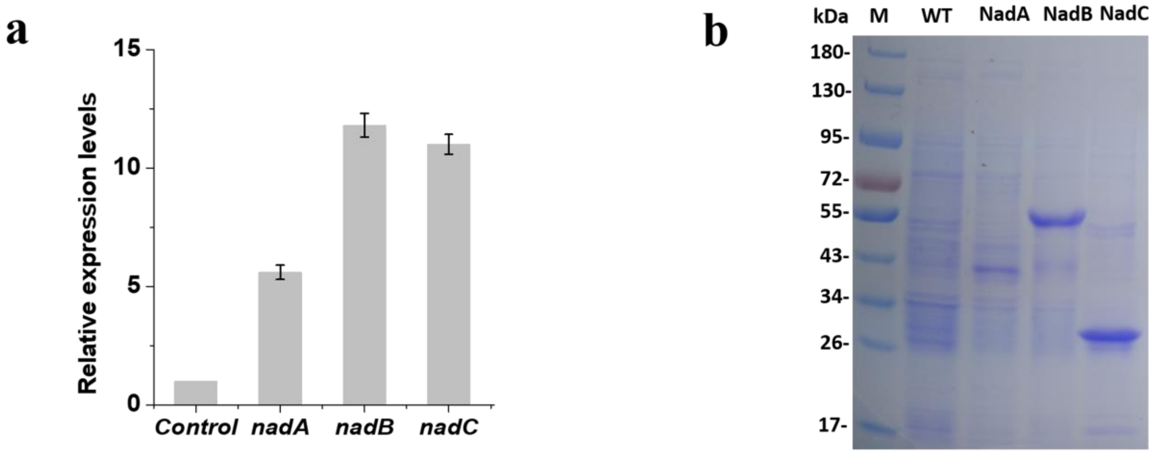

Module 2 - Salvage biosynthesis

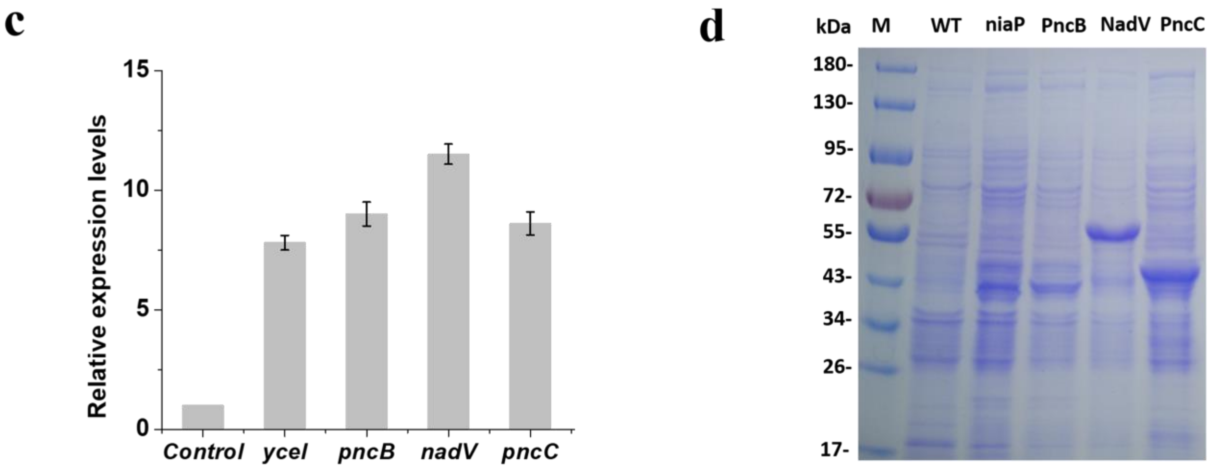

Module 3 – Universal biosynthesis

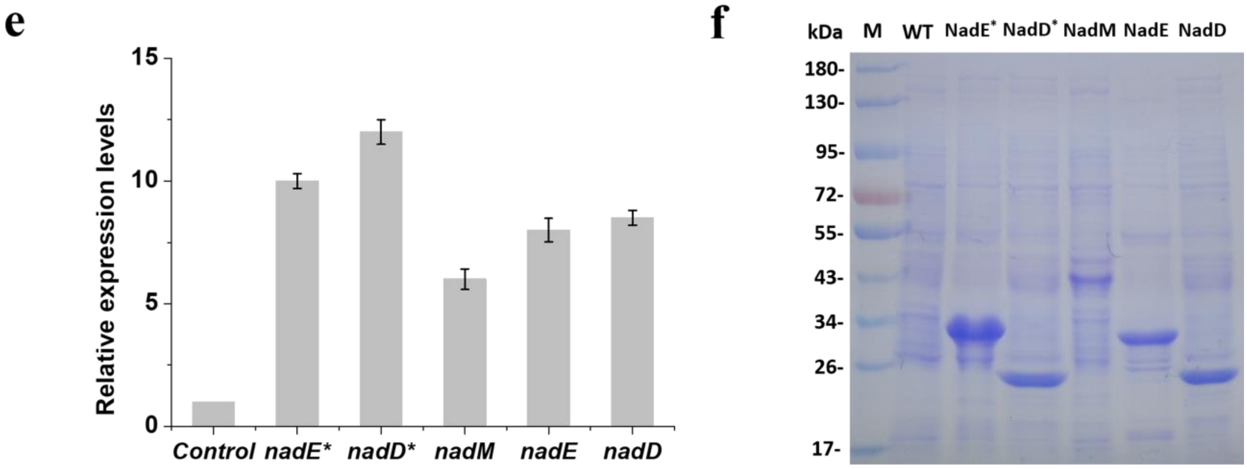

Supplementary Figure 1. The RT-qPCR and SDS/PAGE analyses of all the overexpressed genes. Among them, the genes *nadA*, *nadB*, and *nadC* from the *de novo* biosynthesis pathway (Module 1); genes *ycel*, *pncB*, *nadV*, and *pncC* from the salvage biosynthesis pathway (Module 2); and genes *nadE**, *nadD**, *nadM*, *nadE*, and, *nadD* from the universal biosynthesis pathway (Module 3). (a) A depict of RT-qPCR analysis of genes *nadB*, *nadA*, and *nadC* from strains De novo-1, De novo-2, and De novo-3, respectively. (b) A depict of SDS/PAGE analysis of nadB, nadA, and nadC proteins from the strains De novo-2, De novo-1, and De novo-3, respectively. (c) A depict of RT-qPCR analysis of genes *ycel*, *pncB*, *nadV*, and *pncC* from the strains SalvageNa-1, SalvageNa-2, SalvageNm-3, and SalvageNm-4, respectively. (d) A depict of SDS/PAGE analysis of ycel, pncB, nadV, and pncC proteins from the strains SalvageNa-1, SalvageNa-2, SalvageNm-3, and SalvageNm-4, respectively. (e) A depict of RT-qPCR analysis of genes *nadD*, *nadE*, *nadD**, *nadE**, and *nadM* from the strains Univer-1, Univer-2, and Univer-4, Univer-5, and Univer-7 respectively. (f) A depict of SDS/PAGE analysis of nadD, nadE, nadD*, nadE*, and nadM proteins from the strains Univer-1, Univer-2, and Univer-4, Univer-5, and Univer-7 respectively. The *gyrB* gene was used as the internal control in RT-qPCR. The transcription and translation levels of all the genes were detected upon induction by 1mM IPTG. The error bars represent the standard deviation from three independent experiments.

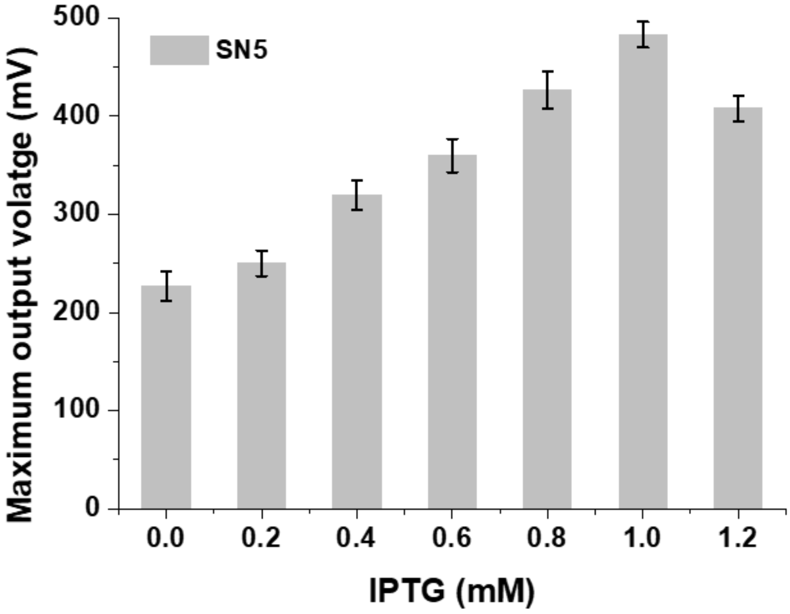

Supplementary Figure 2. The output voltage of recombinant *S. oneidensis* SN5 upon induction by different levels of IPTG. The error bars represent the standard deviation from three independent experiments.

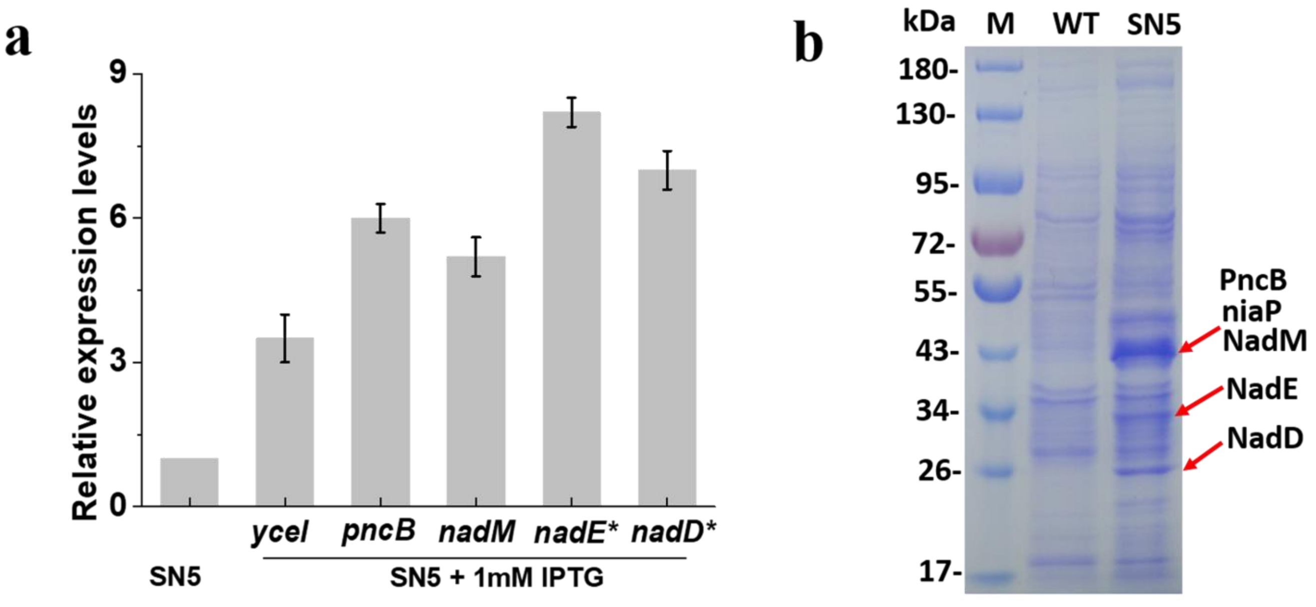

Supplementary Figure 3. The RT-qPCR and SDS/PAGE analyses of all the overexpressed genes in the recombinant *S. oneidensis* SN5 strain upon induction by 1mM IPTG. (a) A depict of RNA transcription levels of genes *ycel*, *pncB*, *nadM*, *nadE**, and *nadD** in the recombinant *S. oneidensis* SN5. The *gyrB* gene was used as the internal control in RT-qPCR. (b) A depict of SDS/PAGE analysis of ycel, pncB, nadM, nadE*, and nadD* proteins in recombinant *S. oneidensis* SN5. The error bars represent the standard deviation from three independent experiments.

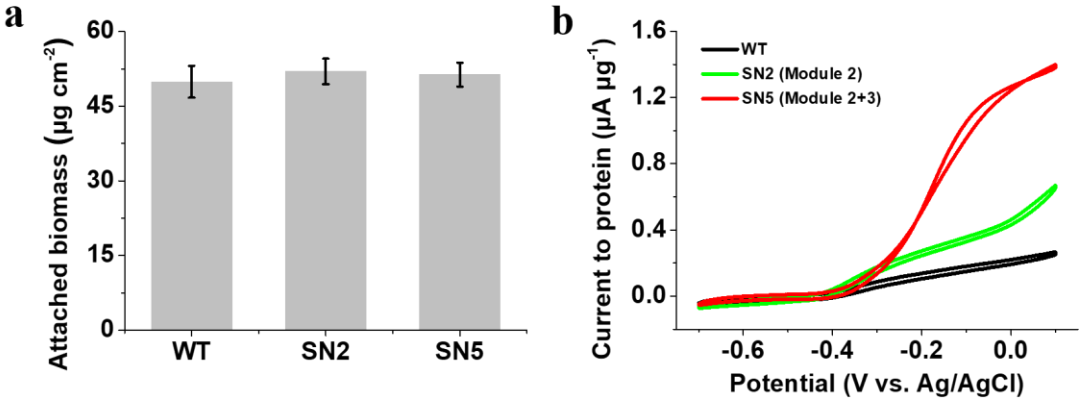

Supplementary Figure 4. Biochemical and bioelectrochemical analyses of the attached biomass on the anode. (a) The attached biomass of each strain on the anodes was measured from three independent MFC anode chambers. (b) Standardized CV curves normalized by the attached biomass on the anodes. The scan rate of CV was 1 mV s^{-1}. The error bars represent the standard deviation from three independent experiments.

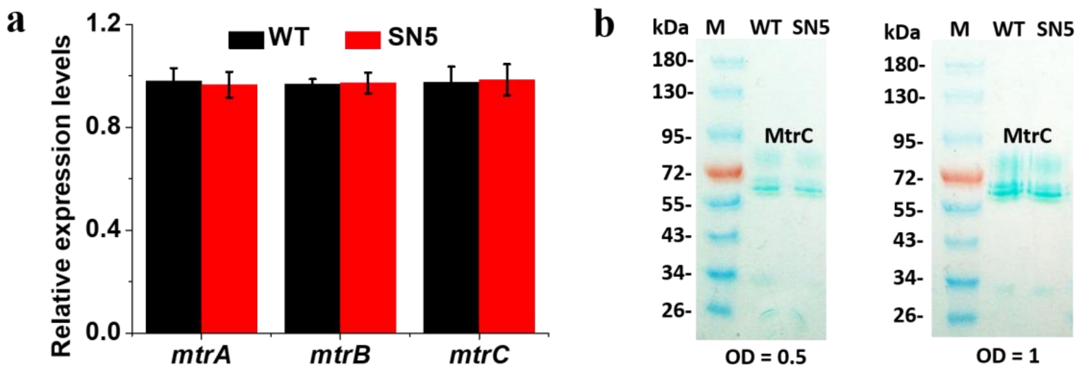

Supplementary Figure 5. Transcriptional and translational levels of MtrCAB complex in the wild-type (WT) and recombinant *S. oneidensis* strain SN5. (a) The relative expression levels of *mtrA*, *mtrB*, and *mtrC* genes in the WT and the recombinant *S. oneidensis* SN5. The error bars represent the standard deviation from three independent experiments. (b) Heme staining of MtrC at OD=0.5 and 1.0.

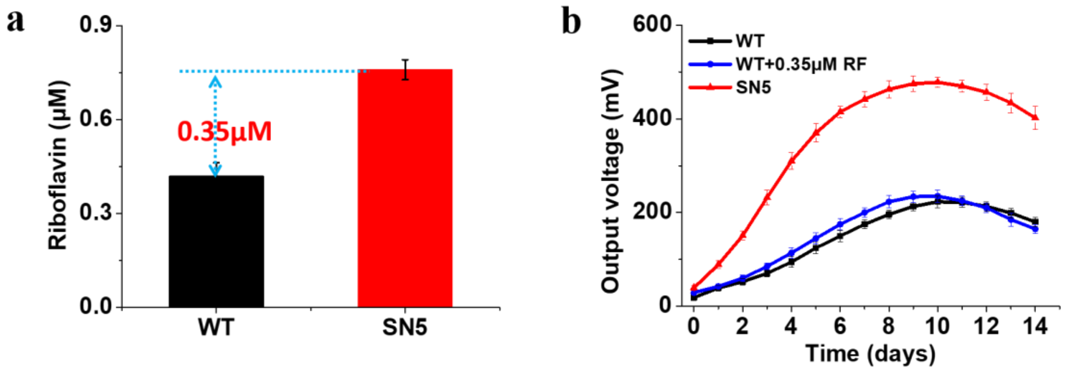

Supplementary Figure 6. The measurement of riboflavin (RF) concentrations and the effect of exogenous addition of additional riboflavin on the output voltage of MFC. (a) Riboflavin concentration was measured from the MFC anode chambers inoculated with the WT and the recombinant *S. oneidensis* strain SN5. The riboflavin concentration synthesized by the recombinant *S. oneidensis* strain SN5 was increased 0.35μM as compared to that of the WT strain. (b) Voltage output in MFCs of the WT and the recombinant *S. oneidensis* strain SN5. The excess 0.35μM riboflavin was added in MFCs of the WT *S. oneidensis* strain to explore the effect of the exogenous addition of riboflavin on its output voltage. The error bars represent the standard deviation from three independent experiments.

Supplementary Table 1. The functional role and source of the overexpressed genes in this study

Module	Gene	Aliases	Functional role	E.C.	Molecular weight (kDa)	Source
De novo biosynthesis (Module 1)	*nadA*	SO_2342	Quinolinate synthetase	EC 2.5.1.72	38.7	*S. oneidensis* [1,2]
	nadB	SO_1341	L-aspartate oxidase	EC 1.4.3.16	59.7	*S. oneidensis* [1,2]
	nadC	SO_0419	Quinolinate phosphoribosyl-transferase	EC 2.4.2.19	31.8	*S. oneidensis* [1,2]
Salvage biosynthesis (Module 2)	*yceI*	BSU02950	Na and Nm niaP transporter	-	43.7	*B. subtilis*[2,3]
	pncB	STM1004	Na phosphoribosyl-transferase	EC 6.3.4.21	45.7	*S. typhimurium*[4,5]
	nadV	SO_1981	Nm phosphoribosyl-transferase	EC 2.4.2.12	55.1	*S. oneidensis* [1,2]
	pncC	SO_0272	Nm mononucleotide deamidase	EC 3.5.1.42	46.2	*S. oneidensis* [1]
Universal biosynthesis (Module 3)	*nadE**	b1740	NAD^+ synthetase	EC 6.3.5.1	30.6	*E. coli* [6,7]
	*nadD**	b0639	NaMN adenylyltransferase	EC 2.7.7.18	24.5	*E. coli* [6,7]
	nadM	FTT_0386	NMN adenylyltransferase	EC 2.7.7.18	40.4	*F. tularensis* [8,9]
	nadE	SO_2021	NAD^+ synthetase	EC 6.3.1.5	30.4	*S. oneidensis* [1,2]
	nadD	SO_1171	NMN adenylyl-transferase	EC 2.7.7.18	24.2	*S. oneidensis* [1,2]

Supplementary Table 2. Synthesized gene sequences in this study

Genes	Sequences
yceI	GCAGAATTCGCGGCCGCTTCTAGAGTACTAGAGAAAGAGGAGAAAT<br>ACTAGATGGGTAAACAACAACCAATCTCTCAACGTAAATTATTAGGT<br>GTTGCTGGTTTAGGTTGGTTATTCGATGCTATGGATGTTGGTATCTTAT<br>CTTTCATCATCGCTGCTTTACACGTTGAATGGAACTTATCTCCAGAAG<br>AAATGAAATGGATCGGTTCTGTTAACTCTATCGGTATGGCTGCTGGTG<br>CTTTCTTATTCGGTTTATTAGCTGATCGTATCGGTCGTAAAAAAGTTTT<br>CATCATCACTTTATTATGTTTCTCTATCGGTTCTGGTATCTCTGCTTTCG<br>TTACTTCTTTATCTGCTTTCTTAATCTTACGTTTCGTTATCGGTATGGGT<br>TTAGGTGGTGAATTACCAGTTGCTTCTACTTTAGTTTCTGAAGCTGTT<br>GTTCCAGAAAAACGTGGTCGTGTTATCGTTTTATTAGAATCTTTCTGG<br>GCTGTTGGTTGGTTAGCTGCTGCTTTAATCTCTTACTTCGTTATCCCAT<br>CTTTCGGTTGGCAAGCTGCTTTATTATTAACTGCTTTAACTGCTTTCTA<br>CGCTTTATACTTACGTACTTCTTTACCAGATTCTCCAAAATACGAATCT<br>TTATCTGCTAAAAAACGTTCTATGTGGGAAAACGTTAAATCTGTTTGG<br>GCTCGTCAATACATCCGTCCAACTGTTATGTTATCTATCGTTTGGTTCT<br>GTGTTGTTTTCTCTTACTACGGTATGTTCTTATGGTTACCATCTGTTAT<br>GTTATTAAAAGGTTTCTCTATGATCCAATCTTTCGAATACGTTTTATTA<br>ATGACTTTAGCTCAATTACCAGGTTACTTCTCTGCTGCTTGGTTAATC<br>GAAAAAGCTGGTCGTAAATGGATCTTAGTTGTTTACTTAATCGGTACT<br>GCTGGTTCTGCTTACTTCTTCGGTACTGCTGATTCTTTATCTTTATTATT<br>AACTGCTGGTGTTTTATTATCTTTCTTCAACTTAGGTGCTTGGGGTGT<br>TTTATACGCTTACACTCCAGAACAATACCCAACTGCTATCCGTGCTAC<br>TGGTTCTGGTACTACTGCTGCTTTCGGTCGTATCGGTGGTATCTTCGG<br>TCCATTATTAGTTGGTACTTTAGCTGCTCGTCACATCTCTTTCTCTGTT<br>ATCTTCTCTATCTTCTGTATCGCTATCTTATTAGCTGTTGCTTGTATCTT<br>AATCATGGGTAAAGAAACTAAACAAACTGAATTAGAATAATACTAGA<br>GTACTAGTAGCGGCCGCCTGCAGG
pncB	GCAGAATTCGCGGCCGCTTCTAGAGTACTAGAGAAAGAGGAGAAAT<br>ACTAGATGACTCAATTCGCTTCTCCAGTTTTACACTCTTTATTAGATAC<br>TGATGCTTACAAATTACACATGCAACAAGCTGTTTTCCACCACTACTA<br>CGATGTTCAAGTTGCTGCTGAATTTCGTTGTCGTGGTGATGATTTATT<br>AGGTATCTACGCTGATGCTATCCGTGAACAAGTTGATGCTATGCAACA<br>CTTACGTTTATTAGAAGATGAATTTCAATGGTTATCTGGTTTACCATTC<br>TTCAAACCAGATTACTTAAACTGGTTACGTGAATTTCGTTACAACCCA<br>GCTCAAGTTTGTGTTACTAACGATAACGGTAAATTAAACATCCGTTTA<br>ACTGGTCCATGGCGTGAAGTTATCATGTGGGAAGTTCCATTATTAGCT<br>GTTATCTCTGAATTAGTTCACCACTACCGTTCTCCAAACGCTGGTGTA<br>GACCAAGCTCTCGATGCTTTAGAGTCTAAATTAGTTGATTTCACTGCT<br>TTAACTGCTAACTTAGATATGTCTCGTTTCCACTTAATGGATTTCGGTA<br>CTCGTCGTCGTTTCTCTCGTGAAGTTCAACAAGCTATCGTTAAACGTT<br>TACAACAAGAATCTTGGTTCGTTGGTACTTCTAACTACGATTTAGCTC

	GTCGTTTAGCTTTAACTCCAATGGGTACTCAAGCTCACGAATGGTTCC<br>AAGCTCACCAACAAATCTCTCCAGATTTAGCTACTTCTCAACGTGCT<br>GCTTTAGCTGCTTGGTTAAACGAATACCCAGATCAATTAGGTATCGCT<br>TTAACTGATTGTATCACTATGGATGCTTTCTTACGTGATTTCGGTATCG<br>AATTTGCTTCTCGTTACCAAGGTTTACGTCACGATTCTGGTGATCCAG<br>TTGCTTGGGGTGAAAAAGCTATCGCTCACTACGAAAAATTAGGTATC<br>GATCCATTAACAAAAACTTTAGTGTTCAGCGATAACTTAGATTTACCA<br>AAAGCTGTTGAATTATACCGTCACTTCGCTTCTCGTGTTCAATTATCTT<br>TCGGTATCGGTACTCGTTTAACTTGTGATATCCCACAAGTTAAACCAT<br>TAAACATCGTTATCAAATTAGTTGAATGTAACGGTAAACCAGTTGCTA<br>AATTATCTGATTCTCCAGGTAAAACTATCTGTCACGATAAAGCTTTCG<br>TTCGTGCTCTCCGTAAAGCGTTCGATTTACCACAGGTTCGTAAAGCTT<br>CTTAATACTAGAGTACTAGTAGCGGCCGCCTGCAGG
*nadD**	GCAGAATTCGCGGCCGCTTCTAGAGTACTAGAGAAAGAGGAGAAAT<br>ACTAGATGAAATCTTTACAAGCTTTATTCGGTGGTACTTTCGATCCAG<br>TTCACTACGGTCACTTAAAACCAGTTGAAACTTTAGCTAACTTAATCG<br>GTTTAACTCGTGTTACTATCATCCCAAACAACGTTCCACCACACCGTC<br>CACAACCAGAAGCTAACTCTGTTCAACGTAAACACATGTTAGAATTA<br>GCTATCGCTGATAAACCATTATTCACTTTAGATGAACGTGAATTAAAA<br>CGTAACGCTCCATCTTACACTGCTCAAACTTTAAAAGAATGGCGTCA<br>AGAACAAGGTCCAGATGTTCCATTAGCTTTCATCATCGGTCAAGATTC<br>TTTATTAACTTTCCCAACTTGGTACGAATACGAAACTATCTTAGATAAC<br>GCTCACTTAATCGTTTGTCGTCGTCCAGGTTACCCATTAGAAATGGCT<br>CAACCACAATACCAACAATGGTTAGAAGATCACTTAACTCACAACCC<br>AGAAGATTTACACTTACAACCAGCTGGTAAAATCTACTTAGCTGAAA<br>CTCCATGGTTCAACATCTCTGCTACTATCATCCGTGAACGTTTACAAA<br>ACGGTGAATCTTGTGAAGATTTATTACCAGAACCAGTTTTAACTTACA<br>TCAACCAACAAGGTTTATACCGTTAATACTAGAGTACTAGTAGCGGCC<br>GCCTGCAGG
*nadE**	GCAGAATTCGCGGCCGCTTCTAGAGTACTAGAGAAAGAGGAGAAAT<br>ACTAGATGACTTTACAACAACAAATCATCAAAGCTTTAGGTGCTAAA<br>CCACAAATCAACGCTGAAGAAGAAATCCGTCGTTCTGTTGATTTCTT<br>AAAATCTTACTTACAAACTTACCCATTCATCAAATCTTTAGTTTTAGGT<br>ATCTCTGGTGGTCAAGATTCTACTTTAGCTGGTAAATTATGTCAAATG<br>GCTATCAACGAATTACGTTTAGAAACTGGTAACGAATCTTTACAATTC<br>ATCGCTGTTCGTTTACCATACGGTGTTCAAGCTGATGAACAAGATTGT<br>CAAGATGCTATCGCTTTCATCCAACCAGATCGTGTTTTAACTGTTAAC<br>ATCAAGGGCGCTGTACTGGCTTCTGAGCAGGCTCTCCGTGAAGCTGG<br>CATCGAATTATCTGATTTCGTTCGTGGTAACGAAAAAGCTCGTGAAC<br>GTATGAAAGCTCAATACTCTATCGCTGGTATGACTTCTGGTGTTGTTG<br>TTGGTACTGATCACGCTGCTGAAGCTATCACTGGTTTCTTCACTAAAT<br>ACGGTGATGGTGGTACTGATATCAACCCATTATACCGTTTAAACAAAC<br>GTCAAGGTAAACAATTATTAGCTGCTTTAGCTTGTCCAGAACACTTAT<br>ACAAAAAAGCTCCAACTGCTGATTTAGAAGATGATCGTCCAAGCTTA

	CCAGATGAAGTTGCTTTAGGTGTTACTTACGATAACATCGATGATTAC<br>TTAGAAGGTAAAAACGTTCCACAACAAGTTGCTCGTACTATCGAAAA<br>CTGGTACTTAAAAACTGAACACAAACGTCGTCCACCAATCACTGTTT<br>TCGATGATTTCTGGAAAAAATAATACTAGAGTACTAGTAGCGGCCGCC<br>TGCAGG
nadM	GCAGAATTCGCGGCCGCTTCTAGAGTACTAGAGAAAGAGGAGAAAT<br>ACTAGATGTACGATATCTCTGTTTTCATCGGTCGTTTCCAACCATTCCA<br>CAAAGGTCACTTACACAACATCATCATCGCTTTACAAAACTCTAAAA<br>AAGTTATCATCAACATCGGTTCTTGTTTCAACACTCCAAACATCAAAA<br>ACCCATTCTCTTTCGAACAACGTAAACAAATGATCGAATCTGATTTAC<br>AAGTTGCTGGTATCGATTTAGATACTGTTGTTATCGAACCATTAGCTGA<br>TTACTTCTACCAAGAACAAAAATGGCAAGATGAATTACGTAAAAACG<br>TTTACAAACACGCTAAAAACAACAACTCTATCGCTATCGTTGGTCAC<br>ATCAAAGATTCTTCTTCTTACTACATCCGTTCTTTCCCAGAATGGGATT<br>ACATCGGTGTTGATAACTACAAAAACTTCAACGCTACTGAATTTCGTC<br>AAAAATTCTACAACGGTATCATCTCTAAACAATACATGTGTTCTAACG<br>ATCCAAAATTAGGTACTTACAACTTCTTAACTAAATTCATGGATACTC<br>AAGTTTACCAAGATTTAGTTGCTGAAAACAACTACGTTATCGAATACA<br>AACGTTTATGGTTAAAAGCTCCATTCAAACCAAACTTCGTTACTGTTG<br>ATGCTTTAGTTATCGTTAACGATCACATCTTAATGGTTCAACGTAAAG<br>CTCACCCAGGTAAAGATTTATGGGCTCTCCCAGGTGGCTTCTTAGAAT<br>GTGACGAAACTATCGCTCAAGCTATCATCCGTGAATTATTCGAAGAA<br>ACTAACATCAACTTAACTCACGAACAATTAGCTATCGCTAAACGTTGT<br>GAAAAAGTTTTCGATTACCCAGATCGTTCTGTTCGTGGTCGTACTATC<br>TCTCACGTTGGTTTATTCGTTTTCGATCAATGGCCATCTTTACCAGAA<br>ATCAACGCTGCTGATGATGCTAAAGATGTTAAATGGATCTCTTTAGGT<br>TCTAACATCAAAAACATCTGTGATCGTATGTTAGAAGATCACTACCAA<br>ATCATCACTATCTTATTAGAAGAATGTGGTAAAAAATTATAATACTAGA<br>GTACTAGTAGCGGCCGCCTGCAGG

Supplementary Table 3. The main constituents of the M9 buffer [10]

Main constituent	Concentration
Na_2HPO_4	6 g l^{-1}
KH_2PO_4	3 g l^{-1}
NaCl,	0.5 g l^{-1}
NH_4Cl	1 g l^{-1}
$MgSO_4$	1 mM
$CaCl_2$	0.1 mM

Supplementary Table 4. Primers used in RT-qPCR

qPCR primer name	Sequences (5’ to 3’)
***nadA*-F**	TCGTCACTTAGGTTCTTACATCGC
***nadA*-R**	AGCATCAGCCATAGCAACAACAG
***nadB*-F**	TAGTTGCTGGTGCTGGTTTATGTG
***nadB*-R**	GTGTAAGATACGACGGTGAGAGTG
***nadC*-F**	TTCTCCCAAACCAAGTGCTCTG
***nadC*-R**	ACCACCACCACAAGTAACAGC
***nadD*-F**	GCGTATCGGTATCTTAGGTGGTAC
***nadD*-R**	GTGTGGTGGGATGTGGTTTGG
***nadE*-F**	TGGTGGTGTTGATTCTTCTACTGC
***nadE*-R**	GGTAAACGAACAGCGATGAATTGG
***nadD**-F**	CGATCCAGTTCACTACGGTCAC
***nadD**-R**	TCTGGTTGTGGACGGTGTGG
***nadE**-F**	TGCTATCGCTTTCATCCAACCAG
***nadE**-R**	CAGTACCAACAACAACACCAGAAG
***nadM*-F**	CGTTCTGTTCGTGGTCGTACTATC
***nadM*-R**	GCGTTGATTTCTGGTAAAGATGGC
***nadV*-F**	GAATCGTGACTTCTTCCAACAACC
***nadV*-R**	GTATACAGAGCCTTCGGGTAAAGC
***pncB*-F**	CGTCGTCGTTTCTCTCGTGAAG
***pncB*-R**	AACCATTCGTGAGCTTGAGTACC
***pncC*-F**	TACCGTTCTTCTATGCCACACATC
***pncC*-R**	TCTTCAGCAACAACAGCAGTACC
***yecl*-F**	TCGTCAATACATCCGTCCAACTG
***yecl*-R**	AACCAAGCAGCAGAGAAGTAACC
***mtrC*-F**	AACGCTTAGAGATCATCACCAACG
***mtrC* -R**	TCGCTAGAACACATTGACCAACC
***mtrB*-F**	ACTATCGCCAAATTGCTACCTACG
***mtrB* -R**	GTTGCTTGAACCTGCTGTTATCC
***mtrA*-F**	ATTCCGATCTTAACAAGCCTAGCG
***mtrA* -R**	CAGCATACCGTCATTCACACTACC
***gyrB*-F**	GGAACGACGGCTACCAAGA
***gyrB* -R**	GTCAACGCACTACGGAAACC

Supplementary References

1. Galeazzi, L. et al. Identification of nicotinamide mononucleotide deamidase of the bacterial pyridine nucleotide cycle reveals a novel broadly conserved amidohydrolase family. *J. Biol. Chem.* **286**, 40365-40375 (2011).
2. Rodionov, D.A. et al. Transcriptional regulation of NAD metabolism in bacteria: NrtR family of Nudix-related regulators. *Nucleic Acids Res.* **36**, 2047-2059 (2008).
3. Sorci, L. et al. Genomics-driven reconstruction of *Acinetobacter* NAD metabolism: insights for antibacterial target selection. *J. Biol. Chem.* **285**, 39490-39499 (2010).
4. Berrios-Rivera, S.J., San, K.Y. & Bennett, G.N. The effect of NAPRTase overexpression on the total levels of NAD, the NADH/NAD^+ ratio, and the distribution of metabolites in *Escherichia coli*. *Metab. Eng.* **4**, 238-247 (2002).
5. Berrios-Rivera, S.J., San, K.Y. & Bennett, G.N. The effect of increasing NADH availability on the redistribution of metabolic fluxes in *Escherichia coli* chemostat cultures. *Metab. Eng.* **4**, 230-237 (2002).
6. Liang, L. et al. Effects of overexpression of NAPRTase, NAMNAT, and NAD synthetase in the NADH biosynthetic pathways on the NADH pool, NADH/NAD^+ ratio, and succinic acid production with different carbon sources by metabolically engineered *Escherichia coli*. *Biochem. Eng. J.* **81**, 90-96 (2013).
7. Liang, L. et al. Regulation of NAD(H) pool and NADH/NAD^+ ratio by overexpression of nicotinic acid phosphoribosyltransferase for succinic acid production in *Escherichia coli* NZN111. *Enzyme. Microb. Technol.* **51**, 286-293 (2012).
8. Sorci, L. et al. Nicotinamide mononucleotide synthetase is the key enzyme for an alternative route of NAD biosynthesis in Francisella tularensis. *Proc. Natl. Acad. Sci. USA* **106**, 3083-3088 (2009).
9. Huang, N. et al. Bifunctional NMN adenylyltransferase/ADP-ribose pyrophosphatase: structure and function in bacterial NAD metabolism. *Structure* **16**, 196-209 (2008).
10. Yong, Y.C., Yu, Y.Y., Zhang, X. & Song, H. Highly active bidirectional electron transfer by a self-assembled electroactive reduced-graphene-oxide-hybridized biofilm. *Angew. Chem. Int. Ed.* **53**, 4480-4483 (2014).
